# The Emerging Role of Senescence in Ocular Disease

**DOI:** 10.1155/2020/2583601

**Published:** 2020-03-09

**Authors:** Parameswaran G. Sreekumar, David R. Hinton, Ram Kannan

**Affiliations:** ^1^The Stephen J. Ryan Initiative for Macular Research (RIMR), Doheny Eye Institute, Los Angeles, CA 90033, USA; ^2^Department of Pathology, Keck School of Medicine of the University of Southern California, Los Angeles, CA 90033, USA; ^3^Department Ophthalmology, USC Roski Eye Institute, Keck School of Medicine of the University of Southern California, Los Angeles, CA 90033, USA; ^4^Stein Eye Institute, Geffen School of Medicine, University of California, Los Angeles, CA 90095, USA

## Abstract

Cellular senescence is a state of irreversible cell cycle arrest in response to an array of cellular stresses. An important role for senescence has been shown for a number of pathophysiological conditions that include cardiovascular disease, pulmonary fibrosis, and diseases of the skin. However, whether senescence contributes to the progression of age-related macular degeneration (AMD) has not been studied in detail so far and the present review describes the recent research on this topic. We present an overview of the types of senescence, pathways of senescence, senescence-associated secretory phenotype (SASP), the role of mitochondria, and their functional implications along with antisenescent therapies. As a central mechanism, senescent cells can impact the surrounding tissue microenvironment via the secretion of a pool of bioactive molecules, termed the SASP. An updated summary of a number of new members of the ever-growing SASP family is presented. Further, we introduce the significance of mechanisms by which mitochondria may participate in the development of cellular senescence. Emerging evidence shows that extracellular vesicles (EVs) are important mediators of the effects of senescent cells on their microenvironment. Based on recent studies, there is reasonable evidence that senescence could be a modifiable factor, and hence, it may be possible to delay age-related diseases by modulating basic aging mechanisms using SASP inhibitors/senolytic drugs. Thus, antisenescent therapies in aging and age-related diseases appear to have a promising potential.

## 1. Introduction

Cellular senescence is the irreversible loss of proliferation potential of somatic cells and a variety of associated phenotypic changes that follow [1]. The concept of cellular senescence stems from pioneering studies showing that human diploid fibroblasts have a finite proliferative capacity in culture, despite the fact that they can stay metabolically active even after entering a stable, nondividing stage [2]. Subsequently, it was shown that senescence could be induced prematurely by many agents. Several independent studies have shown that senescent cells also play a role in multiple biological processes such as embryonic development, wound healing, tissue repair, tumorigenesis, aging, and age-related disease [3]. Thus, studying senescence in the eye and its association with age-related macular degeneration (AMD) will be of great interest. Herein, the nature and role of multiple senescence inducers characterized by an array of multiple biomarkers in use as well as mechanisms of cellular senescence are reviewed. In addition, the role of mitochondria in cellular senescence with special reference to ocular diseases such as AMD is also addressed. Finally, the review summarizes available information on senolytic drugs currently used in animal models and in clinical trials.

## 2. Acute or Chronic Senescence

Given the involvement of the process of senescence in many activities, it raises the question whether processes of the senescent cells involved could be similar or different. Generally, senescence belongs to one of two categories: acute (transient or programmed) or chronic (damage/stress induced) [4, 5]. Such differentiation would allow understanding the dual (beneficial vs. harmful) role of senescence on normal development and regenerative processes, as well as its role in human disease and aging. Developmentally programmed senescence is a normal physiological process of the body that occurs in response to developmental events, whereas damage-/stress-induced senescence is triggered by nonphysiological stimuli or disease stages.

Acute senescence is mostly beneficial and presumably does not contribute to aging; it relies on the coordinated action of senescent cell production and subsequent elimination—the processes involved in wound healing, tissue remodeling, and embryogenesis. Senescence has been demonstrated in the endolymphatic sac and mesonephros of the mouse and human embryos followed by macrophage-mediated removal of senescence cells [4]. Further evidence of senescence was shown in the apical ectodermal ridge and the senescence-associated secretory phenotype (SASP) produced by these cells induces tissue remodeling [6]. Developmental senescence is p21 dependent, but p53 independent, and shares many common features with stress-induced senescence, including a common gene expression signature and senescence-associated *β*-galactosidase activity [4]. These landmark studies revealed that cellular senescence during embryonic development is a programmed, transient event that contributes to tissue remodeling via SASP or to altered cellularity through clearance, suggesting a primordial role in normal physiology.

Paradoxically, while chronic senescence can initially have beneficial effects, its long-term existence could potentially aggravate age-related diseases [7]. “Chronic” senescence develops gradually because of progressive damage over time as seen in aging and age-related diseases. During chronic senescence, the switch from temporal to persistent cell cycle arrest appears to be random, induced by the multiple inducing factors acting simultaneously on a cell. These results in arrest of proliferation and ultimately cells become dysfunctional and most importantly negatively affect local environment by a nonautonomous mechanism [8]. The differential effects of developmental versus pathological senescence on aging could be associated with their effect on other cells via the SASP. Therefore, removal of chronic senescent cells during both premature and normal aging is able to reduce the development and progression of many age-associated dysfunctions [9, 10]. However, the molecular mechanism governing the different types of senescence *in vitro* and *in vivo* is still not fully explored. It is hypothesized that the kinetics and efficiency of senescent cell clearance could be one of the key differences between acute and chronic senescence. Further research will strengthen our understanding of the relationship between acute vs. chronic senescence.

## 3. The Beneficial and Detrimental Role of Senescent Cells

As described earlier, senescence has been shown to have a dual role, beneficial in some contexts and detrimental in others. Senescence acts by tumor suppressor mechanisms and thus inhibits the proliferation of cancer cells and is involved in embryonic development [4, 6], wound healing [11], and tissue repair [12, 13]. Senescent cells are metabolically highly active and actively secrete an array of proinflammatory cytokines and chemokines, growth factors and extracellular matrix degrading proteins, and the SASPs [14]. It is believed that SASP molecules stimulate movement of immune cells to the senescent cells; activate and promote their clearance [12].

The beneficial process of the senescence can be compromised in aged tissues, resulting in the accumulation of senescent cells that could potentially enhance tissue dysfunction through SASP which is particularly rich in proinflamatory cytokines and matrix metalloproteinases [15–19]. Thus, senescence has been linked to aging and age-related diseases. For example, aging human skin has increased numbers of cells that are positive for SA-ß-gal [20]. In aged cells, the components of SASP has been shown to activate neighboring healthy cells to senescent cells [21]. It is of utmost interest to learn why these senescent cells in aging are not eliminated as is the case for embryonic cells and the inefficient immune system may play a role in this regard.

## 4. The Phenotype Associated with Cellular Senescence

Senescent cells are featured by an array of specialized features which have been extensively reviewed [3, 22, 23]. Since senescent cells are widely heterogeneous and some of their features are common in other nonsenescence cellular states, it is difficult to clearly identify senescent cells using a few markers [22, 24]. The following are some of the phenotypes associated with senescent cells:

- Senescence is generally accompanied by significant morphological alterations. The senescent cells become flat and enlarged more than double the size of nonsenescent cells due to rearrangement of the cytoskeleton, particularly vimentin filaments [25, 26]
- Senescence-associated heterochromatin foci (SAHF) are specialized domains of facultative heterochromatin observed in the nuclei of senescent cells as punctate DNA-stained dense foci [27]. Specifically, SAHF are not associated with nonsenescent or quiescent state
- *Senescent cells* normally display increased activity of the acidic *senescence*-associated *β*-*galactosidase* (SA-*β*-*gal*), which partly reflects the increase in lysosomal mass [20, 28]
- Most senescent cells are characterized by the increased expression of antiproliferative molecules (p16INK4a). Increased expression of p16INK4a with age in mice and humans has been well documented [29–31]
- Senescent cells secrete cytokines, chemokines, extracellular matrix proteases, and growth factors, collectively known as SASP or senescence-messaging secretome [32, 33]
- Senescent cells are characterized by significant changes in mitochondrial morphology, function, and metabolism. Senescent cells remain metabolically active and have increased mitochondrial biogenesis and respiration [34]

## 5. Biomarkers of Senescence

**Figure 1:**
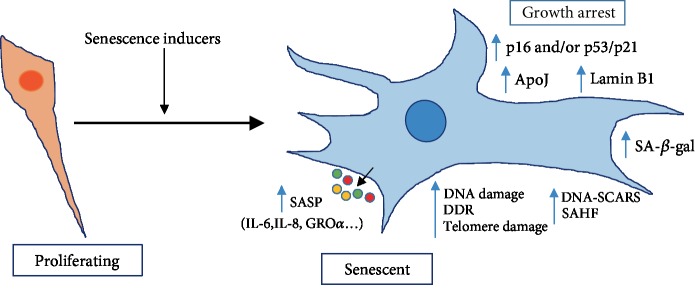
Schematic representation of senescence markers. Cellular senescence can be induced by multiple agents (senescence inducers). The senescent cell is morphologically different and bigger in size. Senescent cells also have increased levels of factors (right) which are used as markers. Senescent cells produce and secrete a complex combination of factors, collectively referred as the senescence-associated secretory phenotype (SASP). SA-*β*-gal: senescence-associated beta-galactosidase; ApoJ: apolipoprotein J; SAHF: senescence-associated heterochromatin foci; DDR: DNA-damage response; DNA-SCARS: DNA segments with chromatin alterations reinforcing senescence. This is adapted from [37].

As described above, no single characteristic is exclusive to the senescent state. Nor do all senescent cells display all the senescence markers identified so far. The identification of senescent cells in vivo is challenging, especially considering their diversity and heterogeneity. Therefore, senescent cells are generally identified by an array of characteristics. Though an array of senescence markers has been proposed and widely used in multiple cell types, no single one can reliably identify senescent cells either *in vitro* or *in vivo*. The most widely used markers of senescence include the senescence-associated *β*-galactosidase (SA-*β*-gal) reactivity [15, 20, 35–39], increased expression of the cyclin-dependent kinase (CDK) inhibitor p16INK4a [15, 30, 36–40], p21 (CIP1/WAF1) [36–39, 41, 42], p53 [36, 37, 41, 42], induction of SASP factors [14, 41, 43–50], mitochondrial DNA modifications [39, 50–57], and chromatin modifications [37, 47, 58, 59] (Figure 1). The limitation is that even a combination of multiple markers does not truly represent senescence but could also describe long-term cell arrest. That is one of the major reasons for intensive research in pursuit of additional robust and sensitive markers.

## 6. Models of Aging in *In Vitro* Systems

In response to cellular stress or damage, proliferating cells enter in a state of long-term cell cycle arrest. Senescence can be induced in cell culture by using multiple stimuli with known common agents. The known models of senescence are summarized in Table 1.

### 6.1. Replicative Senescence (RS)

With the exception of tumor cells and some stem cells, replicative senescence appears to be a fundamental feature of somatic cells [60]. The first formally described senescence model [2] refers to an irreversible arrest of cell proliferation and altered cell function following multiple cell divisions [61]. Telomere shortening because of multiple cell passages has been considered a plausible mechanism [23, 61]. Replicative senescence is characterized by DNA double-strand breaks, with activated ataxia telangiectasia mutated (ATM), and/or ATM and RAD3-related (ATR) mechanisms and their respective mediators, checkpoint kinase 2 (CHK2) and CHK1 [62].

### 6.2. Stress-Induced Premature Senescence (SIPS)

Cellular senescence can be induced by various stresses including oxidative stress. Both chronic and acute oxidative stress protocols were used to induce cellular senescence. Hydrogen peroxide or tert-butyl hydroperoxide is widely used to produce oxidative stress-induced premature senescence within a short period of time [38, 39]. Such induced premature senescent cells display markers that are similar to those from replicative senescent cells. There are many agents that could be used for SIPS, the major classes are cytokines, oxidizing agents, hyperoxia, and copper. Although oxidizing agents work partly through DNA damage, other cellular components such as mitochondria are also affected. In the acute protocol, a single stress dose will inhibit the growth of a fraction of cells with others more or less unharmed. But in the chronic protocol, cells are exposed to repeated stresses, with a dose every day or every other day, allowing to minimize unharmed cells and increasing chances of cells undergoing senescence [63–65]. Thus, SIPS could be a good model to study heterogeneity of aging at the cellular level.

### 6.3. DNA Damage-Induced Senescence (DDR)

Depending on the treatment protocol and dose, DNA damage response (DDR) can induce senescence [66]. Different types of DNA-damaging agents including cancer drugs such as bleomycin or doxorubicin are used to induce this type of senescence. The DNA damage response is initiated by the activation of the PI(3)K- (phosphatidylinositol-3-OH-kinase-) like kinases ATM, ATR (ATM and RAD related), and DNA-PKcs (DNA-dependent protein kinase catalytic subunit) [62]. These kinases also halt progression through the stages of cell cycle by activating the effector kinases (checkpoint kinase) CHK2 and CHK1 [62]. Finally, checkpoint enforcement results from multiple signaling pathways such as p53 and the cell division cycle 25 phosphatases. Slower p53 induction upon phosphorylation by DDR kinases leads to its stabilization and enhanced ability to induce the transcription of p21 which results in a stable cell cycle arrest [62, 67].

### 6.4. Oncogene-Induced Senescence (OIS)

Oncogene activation is a hallmark of cancer; however, oncogene activation in a normal cell induces cellular senescence [68]. Oncogene-evoked senescence was first discovered following expression of an oncogenic form of *Ras* in normal human fibroblasts [69]. This process resembles replicative senescence. A sustained antiproliferative response is due to oncogenic signaling resulting from mutation of an oncogene or the inactivation of a tumor-suppressor gene. The activation of oncogenes, such as Ras or B-Raf proto-oncogene, serine/threonine kinase (BRAF), or the inactivation of tumor suppressors, such as PTEN, will result in OIS [23, 70, 71]. Specifically, OIS is established independently of any telomere attrition or dysfunction [72]. So, atypical activation of signaling pathways and positive cell cycle regulators may lead to the buildup of DNA damage, and the resulting cellular senescence [72].

### 6.5. Mitochondrial Dysfunction-Associated Senescence (MiDAS)

The role of mitochondrial damage in cellular senescence has been well characterized using cell cycle arrest as the main marker of senescence [73]. Very little information about the secretary phenotype in conjunction mitochondrial damage-induced senescence is available. However, a recent study describes senescence induced by multiple types of mitochondrial dysfunction lacks a wide number of cytokines secreted during canonical senescence such as IL-1*α*, IL-1*β*, IL-6, and IL-8 [74]. This secretory phenotype, controlled by NAD-AMPK-p53 axis, appears to be characteristic of this type of senescence [74]. In addition, it was demonstrated that canonical triggers of cellular senescence, when combined with mitochondrial dysfunction, results in a senescence response that also lacks the abovementioned cytokines, suggesting that mitochondrial damage is superior to nuclear damage regarding the inhibition the cytokines [74].

### 6.6. Epigenetically Induced Senescence (EIS)

DNA methylation changes and histone modification have been observed during cellular senescence. Accordingly, treating cells with epigenetic modifiers that inhibit DNA methyltransferases (5-aza-2′-deoxycytidine), or histone deacetylases (sodium butyrate, trichostatin A) or histone acetyltransferases (curcumin, C646), and histone methyltransferases (BRD4770) are also known to cause senescence [75].

## 7. Cellular Senescence during Ocular Aging and Age-Related Diseases

Aging is considered one of the most obvious predisposing factors for the development of AMD because prevalence of this disease rises in those over 60. With aging, the human retina undergoes various structural and physiologic changes [80]. Several independent studies suggest senescence contributes to the development of many ocular diseases (Table 2). Aging has been associated with fewer retinal neurons along with numerous age-related quantitative alterations such as decreased areas of dendritic and axonal arbors and decreased density of cells and synapses [81]. One study found that retinal pigment epithelium (RPE) cells were lost in large numbers in the periphery of the human retina [82] while a second study reported overall RPE to photoreceptor ratio dropped with age throughout the retina [83]. Furthermore, protein levels of canonical senescence markers such as p16, p21, and p53 were shown to increase in the RPE isolated from aged human donors (84-86 years) [84].

RPE cells show signs of senescence when grown *in vitro* for a prolonged period [85]. SA-*β*-gal positivity of RPE cells has also been reported in the human retina and monkey retina [86]. Neurons, but not neuroglia and blood vessels undergo cellular senescence in the aged human retina [42]. Aging causes loss of retinal neurons, including rod photoreceptors, retinal ganglion cells (RGCs), and rod bipolar cells [82, 86, 87]. In addition, intracellularly, lipofuscin deposits in the RPE, which could be a potential inducer of reactive oxygen species (ROS) after exposure to oxygen and light [88]. Retinal microaneurysms overexpress canonical senescence markers, suggesting that cellular senescence is associated with the pathogenesis. Apoptosis also cooccurs with cellular senescence in old-age retinal microaneurysms [42]. The age-related decrease in the anterior segment outflow is largely responsible for the elevated intraocular pressure, one of the factors attributing to the development of glaucoma [89]. Markers of cellular senescence are found in the trabecular meshwork of patients with primary open-angle glaucoma and aging of these cells leads to their decreased function and a consequent decreased outflow facility [90]. The decreased outflow with age is also characterized by a decreased population of RGCs, neuronal cells in the retina that electrically couples the retina with the brain [90, 91]. In the mouse model of acute glaucoma, it has been shown that p53, another key player in cellular senescence [22], contributes to RGC death, as evidenced by protection of RGC in mice lacking p53 [92]. In addition, increased expression of SASP, upon acute glaucoma-induced retinal damage, was also observed suggesting that the senescence-associated cytokine network is activated in IOP-treated retinas [92]. Genome-wide association studies (GWAS) have indicated that the SIX1-SIX6 and P16/INK4A loci are among the strongest risk genes associated with primary open-angle glaucoma (POAG) [93, 94]. Deploying a combination of genetic association and functional studies, Skowronska-Krawczyk et al. [95] demonstrated that SIX6 risk variant upregulates p16/INK4A expression resulting in RGC senescence in cell culture, animal models, and human glaucoma retinas. Beyond loss of retinal cells, aging is also associated with the accumulation of both intracellular and extracellular deposits where it can generate ROS after exposure to oxygen and light [96]. The finding that A*β* is also elevated in aging retina and is a component of drusen suggests that A*β* may be a key factor in AMD pathology and this has opened new perspectives about the potential etiology and therapeutic approaches [97, 98]. A*β* has been recently shown to induce RPE cells to enter senescence [99]. A recent study shows the role of RPE senescence in the retinal degeneration induced by A*β* (1-42) peptide as characterized by upregulation of senescence markers and abnormal electroretinography (ERG) responses [100]. Hence, cellular senescence of RPE or neuronal cells induce different age-related retinal diseases and targeting them could be a viable therapeutic strategy.

## 8. Cell Senescence Signaling Pathways

Many molecules can work alone or in combination and make the cells senescent via p16INK4a/Rb (retinoblastoma protein), p53/p21, and likely other pathways. Cellular senescence is a state of stable cell cycle arrest and the onset and maintenance of the senescent state involve action of two major pathways, the p16Ink4a/Rb pathway and the p19Arf/p53/p21Cip1 pathway [111]. The p16Ink4 is a CDK (cyclin-dependent kinase) inhibitor that accumulates in the cell as the number of cell divisions increase. Independent of the p53/p21Cip1 pathway, the p16Ink4a/Rb pathway is considered to be the primary pathway leading to the development of senescence in cells [112]. Cellular senescence induced by oxidative stress is mainly through the p16Ink4a/Rb pathway. Oxidative stress enhanced the nuclear expression of p16 which can bind to CDK4/6, thus inhibiting the phosphorylation of the retinoblastoma protein Rb and prevent the transcription factors E2F from activating, thereby inhibiting the expression of its regulatory site genes. The mechanism of cells entering the cell cycle from G1 stage to the S stage is prevented, thus inhibiting cell proliferation, and ultimately leading to cell senescence.

Telomere-damaged senescent cells function mainly through the p53/p21Cip1 pathway. When cells are stressed, they can cause high expression of p16 protein. This can lead to the phenotype associated with cell senescence and growth arrest; formation of senescence-associated heterochromatin sites and maintenance of the p16Ink4a/Rb pathway is no longer required. p53 is a transcription factor involved in the regulation of an array of cellular processes, including metabolic adaptation, DNA repair, cell cycle arrest, apoptosis, and senescence (see Bourgeois and Madl, [113] for a review). Several independent studies using p53 inhibitors, p53 antisense oligonucleotides, or homozygous deletion mutants demonstrated a role for p53 in cellular senescence [114]. The p53 protein in the p53/p21Cip1 pathway is a common tumor suppressor protein, which is inactivated in many tumors and upregulated in senescent cells. The ubiquitin ligase MDM2 (murine double minute 2) can promote the degradation of p53 by related proteases or directly inhibit the activity of p53 protein. The p19Arf protein can bind to and inhibit MDM2 activity. When there is damage in the DNA molecule, the upregulated p19Arf protein inhibits MDM2 and activates p53. The p53 then activates downstream p21Cip, inhibits RB phosphorylation, and thus cannot bind to E2F. Blocking cell cycle at the G1 phase makes the cells enter into the senescence state.

## 9. Cellular Senescence and the Role of SASPs

Multiple stressors induce senescence, some even with shared effects that can also drive multiple phenotypes and pathologies associated with aging. Many studies have provided clear evidence that an array of bioactive molecules are released by senescent cells, called SASP [14]. As mentioned earlier, these include chemokines, cytokines, metalloproteases, and growth factors which can act through both autocrine/paracrine pathways and can affect neighboring cells [115]. Although SASP is generally considered proinflammatory, the true microenvironmental impact and composition of SASP may vary according to cell types (i.e., fibroblasts/epithelial, normal/cancerous) and senescence-triggering stimuli (i.e., replicative senescence, DNA damage-induced senescence, oncogene-induced senescence) [14, 116, 117]. It is now evident that SASP functionally links senescence to various biological processes including tissue regeneration and remodeling, embryonic development, inflammation, and tumorigenesis. Based on the mechanism of action, SASP factors can be classified [118] as (1) *receptor mediated*, which includes interleukins IL-6, IL-8, and IL-1*α*; chemokines GRO*α*, GRO*β*, CCL-2, CCL-5, CCL-16, CCL-26, and CCL-20; and the growth factors VEGF, HGF, FGF, TGF-*β*, and GM-CSF [119, 120]; (2) *regulatory molecules* such as tissue inhibitors of metalloproteases (TIMP), the plasminogen activator inhibitor (PAI), and insulin-like growth factor-binding proteins (IGFBP); (3) *directly acting* such as matrix metalloproteases MMP-1, MMP-10, MMP-3 and serine proteases: the tissue plasminogen activator (tPA) and urokinase plasminogen activator (uPA). Several in vitro and in vivo studies have attributed the multifunctions of the SASP to individual protein components. For example, out of the SASPs, IL-6, IL-8, and CCL2 enhance tumor cell proliferation [14, 121]; VEGF promotes angiogenesis [122]; IL-6, IL-8, IGFBP7, and PAI-1 augment senescence [119–124]. It was reported that the multifunctional cytokines such as TGF-*β* family ligands, CCL2, and VEGF evoke cellular senescence [125] while PDGF-AA promotes wound healing [11, 126]. In senescent cells, many of the SASP factors discussed above are activated at the transcriptional level [119, 120, 127]. However, many SASP bioactive factors are able to induce inflammation, disrupt tissue architecture, and enhance malignant transformation [128, 129]. Of note, the inhibition of the SASPs such IL-6 or IL-8 only partially blocks paracrine senescence progression suggesting the involvement of alternate pathways [130, 131].

Recently, increasing evidence suggests that extracellular vesicles (EVs) released from senescent cells are unique and are involved in regulating the phenotype of recipient cells the same way as SASP bioactive molecules [132–136]. Hence, the EVs secreted from senescent cells, (senescence-associated EVs), appear to be a novel SASP factor [137–140].

There are reports demonstrating increased EV secretion and changes in the compositions of EVs with stress [141–145]. Further, the expression of the exosome markers CD63 and LAMP2 showed a significant increase in older retinal pigment epithelium tissue [146]. It is also known that the secretion of EVs increased in human RPE cells rendered senescent by the DNA-damaging agent doxorubicin [144]. However, not much is known about the role that EVs play as SASP mediators in the senescent microenvironment. The miRNA components of senescent exosomes are studied in some cell types but, aside from protumorigenic effects [134], the proteomic content and function of exosomes and small EVs secreted by senescent cells are not well studied. A comparative proteomic analysis of EVs secreted from control and DXR-induced senescent RPE cells found that EVs secreted from senescent cells showed a markedly altered protein composition [144]. Paracrine senescence via the SASP has been previously described as an important mechanism during senescence [125, 147], although these studies do not differentiate between the effect of soluble factors secreted by the cells and EVs released. Furthermore, a recent study in human primary fibroblasts provides evidence that both the soluble factors and sEVs are responsible for mediating paracrine senescence [148]. An in-depth mass spectroscopic analysis of the published protein composition of soluble factor of senescence [125] and sEV proteomics [148] shows little correlation between both fractions, suggesting that although the downstream signaling is similar, the triggers inducing senescence are diverse. A comprehensive proteomic database of soluble and exosome SASP factors (SASP Atlas), originating from multiple senescence inducers and cell types has appeared recently [149]. The protein cargo of exosomes/EVs released by senescent cells was significantly higher and many protein markers will likely be specific to cell type and originating stimulus when compared to quiescent control cells [149]. Mitsuhashi et al. [150] reported that the levels of IL-6 and IL-12 mRNAs in macrophage-derived exosomes from older subjects are higher than those from younger subjects. Furthermore, there is evidence that miRNAs that can regulate cellular senescence are contained in EVs [151, 152]. In addition, mounting evidence suggests that senescence-associated EVs are involved in pathology as well as senescent cells can impact age-related stem cell dysfunction via EVs [132, 143, 145, 153].

## 10. Role of Mitochondrial ROS in the Induction of Senescence

It is well known that both intracellular and extracellular ROS have been shown to contribute to the induction of senescence. Mitochondria produce ROS as a byproduct of electron leak along the electron transport chain during cellular respiration [154]. Hydrogen peroxide (H_2_O_2_), which is a major endogenous ROS, is a potent inducer of cellular senescence. Work from our laboratory has documented that H_2_O_2_ treatment induced senescence in human RPE cells [38, 39]. ROS have also been shown to act as signaling molecules during senescence, stabilizing the cell cycle arrest. ROS contributes to initiating cellular senescence and progression by either directly damaging mitochondrial DNA (mtDNA) or in interaction with modifications of the telomerase reverse transcriptase enzyme, a catalytic subunit of the enzyme telomerase and the p53 and Ras pathways activity [21]. Dysfunctional mitochondria release multiple damage-associated molecular patterns (DAMPs), such as ATP, ROS, and mtDNA, activating the NLRP3 inflammasome, which in turn trigger the proteolytic maturation of proinflammatory cytokine precursors, such as IL-1*β*, and leads to the activation of the NF-*κ*B pathway, supporting senescence biogenesis [155]. In contrast, interventions which reduce mitochondrial ROS such as nicotinamide mononucleotide (NAD) [156] and mitochondrial-targeted antioxidant MitoQ [157, 158] have been shown to prevent telomere dysfunction and prevent senescence. NAD treatment via intravitreal administration in mice also preserves NAD+ and prevents RPE senescence [156]. We have demonstrated that H_2_O_2_-induced senescence altered mitochondrial functions and treatment with a mitochondria-derived peptide, humanin, prevented mitochondrial ROS and delayed cellular senescence [39]. ROS also plays a role as a signaling factor in downstream senescence effector pathways [159]. In addition, canonical markers regulating major senescence pathways such as p16, p21, and p53 are elevated in response to increased ROS [39, 160] suggesting that ROS in senescent cells enhanced the cell cycle arrest characteristic of the senescence phenotype [159]. On the other hand, it is suggested that mitochondrial ROS generation may not necessarily be the primary cause of cellular senescence [161]. The senescence phenotype induced by hyperoxia was not blocked either in the mitochondrial SOD2 or catalase overexpressed human lung fibroblasts suggesting the ROS formed in the cytosol alone can induce senescence [161]. Of note, mitochondrial ROS can damage nuclear DNA and thus can induce senescence [159]. Depletion of mitochondrial DNA (mtDNA), knockdown of mitochondrial sirtuin-3 (SIRT3), or inhibition of the electron transport chain can induce mitochondrial dysfunction-associated senescence (MiDAS) [74]. It was reported that in MiDAS, pyruvate, but not an antioxidant, prevented MiDAS [74]. Further, no evidence of DNA damage was observed but rather decreased NAD+/NADH ratios caused by MiDAS [74].

## 11. Energy Metabolism and Cellular Senescence

Mitochondria are the principal cellular organelles responsible for ATP production, calcium regulation, biosynthetic processes, and apoptotic regulation. It is well established that not only cell size but also mitochondrial mass increases significantly in senescent cells (Table 3). Senescent cells have been observed in the eye specifically in the neuronal, endothelial, and RPE cells [42, 86] and it is critical to understand their participation in energy metabolism during aging as well as the progression of various retinal degenerative diseases.

A strong link between mitochondrial metabolism and the senescent state has been proposed [162]. Acute oxidative stress can cause increased ROS production which is linked to mitochondrial oxidative damage, a reduction in mitochondrial copy number, and decreased mitochondrial respiration and ATP production in RPE cells [39, 163–165]. Repeated exposure stress can also induce ROS which in turn can induce and regulate cellular senescence and can cause major changes in the metabolome [166]. In particular, an increase in mitochondrial oxygen consumption and oxidative phosphorylation have been reported in oncogene-induced senescence in human fibroblasts [166, 167], oxidative stress-induced senescence [50, 168], therapy-induced senescence in lymphoma [169], and DNA damage-induced senescence [50]. However, in replicative senescence, an impairment of mitochondrial function and increased glycolysis has been described [50, 170]. Thus, it can be hypothesized that senescent cells follow different bioenergetic phenotypes, depending on the stimuli which trigger senescence induction. In particular, replicative senescence in primary human mammary epithelial cells is accompanied by a marked inhibition of nucleotide synthesis without any alteration in glycolysis. These findings demonstrate that inhibition of nucleotide synthesis plays a causative role in the establishment of replicative senescence [171].

## 12. Mitochondrial Retrograde Signaling and Senescence

Mitochondrial-to-nuclear signaling has gained much attention in recent years. Retrograde signaling is a mitochondrial-to-nuclear signal transduction pathway by which defective mitochondria communicate with the nuclear genetic compartment [178, 179]. When the oxidative and metabolic activities of mitochondria are altered, it communicates with the nucleus via mitochondrial retrograde signaling. An evidence for this has been reported in mitochondrial damage-associated conditions such as neurodegeneration and cardiovascular diseases [180, 181].

**Figure 2:**
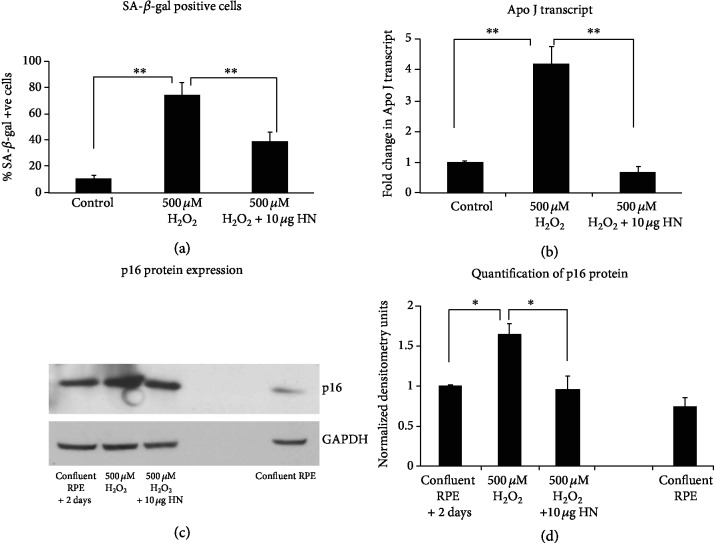
Evidence for increased senescence with oxidative stress and its elimination in human RPE cells with a mitochondria-derived peptide, humanin. ^∗^*P* < 0.05, ^∗∗^*P* < 0.01. Reproduced from [39] and is licensed under a Creative Commons Attribution-NonCommercial-NoDerivatives 4.0 International License.

The retrograde signaling involves multiple factors [181] and these factors mainly activate cytosolic mediators through interactions with small molecules (e.g., Ca2+, ROS, NAD/NADH ratio) and transmit signals into the nucleus [182, 183]. These mitochondrial signals modulate the gene expression of transcription factors, (NRF1, NRF2, Sirt1, mTOR, PPAR*γ*, Sp1, CREB) and members of the PGC-1 family of regulated coactivators (PGC-1*α*, PGC-1*β*, and PRC) [184]. These reprogrammed transcripts could restore mitochondrial function, activate alternative energy pathways, and prepare the cells for death, senescence, or proliferation [181, 185]. Thus, retrograde signaling-mediated transcriptional reprogramming could play a key role in senescence and aging. Increasing evidence indicate that mitochondrial short open reading frame- (sORF-) derived peptides are potent and evolutionarily conserved mitochondrial signals could affect various physiological processes. In support, we have demonstrated that the first mitochondria-derived peptide humanin, transcribed and translated as short ORFs from the 16S rRNA of mtDNA, delayed oxidative stress-induced senescence in RPE cells (Figure 2) [39]. Whether this MDP has a role in the regulation of nuclear factors needs to be studied. However, it has been shown that another MDP, MOTS-c, translocates to the nucleus and regulates nuclear gene expression following metabolic stress via AMPK pathway [186]. Thus, between the nucleus and the mitochondria, signaling occurs in both directions, and further studies of these pathways would provide in-depth details about senescence and organismal aging [187].

## 13. Mitochondrial Dynamics and Cellular Senescence

Mitochondrial dynamics, in general, include events such as fission, fusion, and mitophagy [188, 189]. Senescent cells are metabolically active and have the ability to self-integrate and remodel their morphology [189]. Fission and fusion help mitochondria to regulate cellular energy levels, and a regulated balance among these events ensure normal mitochondrial and cellular functions [190]. In particular, fusion and fission proteins MFN1, MFN2, dynamin-related protein 1 (DRP1), mitochondrial fission factor (MFF), and mitochondrial fission 1 protein (FIS1) modulate mitochondrial shape in response to cellular requirements [191]. The mitochondrial fusion is primarily regulated by Mitofusion proteins 1/2 (Mfn1/2), optic atrophy protein 1 (Opa1) [192, 193], and fission by dynamin-related protein 1 (Drp1) and fission 1 protein (Fis1). We have shown that in oxidative stress-induced RPE senescence, mitochondrial fission proteins, FIS1, and DRP1 increased significantly [194]. Altering mitochondrial dynamics including morphology can cause mitochondrial defects and dysfunction could result in cellular senescence [195, 196]. However, additional studies are required to establish the link between mitochondrial morphology, dynamics, and cellular senescence.

## 14. Mitochondrial Biogenesis and Senescence

It is now appreciated that senescence is accompanied by increased mitochondrial oxidative metabolism, along with increased mitochondrial mass due to increased mitochondrial biogenesis [159, 167, 194, 197, 198]. Mitochondrial biogenesis is a multifactorial process which also involves assembly as well as replication of mtDNA [199]. Despite the complexity of the different signaling pathways that potentially regulate mitochondrial biogenesis, they all use the key component of the peroxisome proliferator-activated receptor *γ* coactivator-1*α* (PGC-1*α*) [200]. The gene expression of nuclear respiration factors (NRF-1 and NRF-2) and mitochondrial transcription factor A (TFAM), which are transcription factors that initiate the expression of both nuclear subunits of the respiratory chain and proteins involved in mitochondrial DNA transcription and replication, are increased by PGC-1*α* [162, 184, 201]. The mitochondrial mass and the mRNA levels of PGC1*α* and NRF-1 were found to increase during replicative senescence [202] and this could be attributed to de novo synthesis of the nuclear transcriptional factors as a compensatory response to increased ROS production and the impaired membrane potential [202]. Overexpression of the PGC-1*α* in human fibroblasts resulted in an increase of the mitochondrial encoded marker protein COX-II, consistent with the ability of PGC-1 to increase mitochondrial number, and accelerated the rate of cellular senescence [202, 203]. In an idiopathic pulmonary fibrosis model, mammalian target of rapamycin/peroxisome proliferator-activated receptor-*γ* complex 1*α*/*β* (mTOR/PGC-1*α*/*β*) axis is markedly upregulated in senescent lung epithelial cells [198]. Despite significant function of PGC-1*α* in mitochondrial biogenesis and senescence, only very few studies are found on its role in the retina, though some studies on its role in biogenesis exist [204, 205]. It has been also demonstrated that PGC-1*α*-deficient mice developed some abnormalities in RPE cells that were associated with their accelerated senescence [200, 206, 207]. A recent study using human ARPE19 cells reported that elevated PGC-1*α* is essential for maintaining normal autophagic flux [206]. Therefore, more detailed studies are required to establish a direct link for PGC-1*α* and senescence in AMD pathology.

## 15. Senolytic Drugs for Targeting Senescent Cells

Because of the adverse nature of senescence in the development of multiple illnesses, disrupting or preventing senescence can delay health decline during aging. The discovery of senotherapeutic drugs represents a developing and highly promising field of current research for new therapies. Inhibition of major pathways or disruption of p53 and p16, p21, have all been shown to have a significant benefit in aging phenotypes but they can increase the occurrence of cancer [208–211]. Therefore, selective removal of senescent cells could be the safer route to target senescence. Senolytics are drugs that selectively target senescent cells, not a molecule or a single biochemical pathway, by inducing apoptosis of senescent cells [212]. Effective use of potential senolytics of natural or synthetic molecules that target fundamental aging processes into clinical practice could be transformative. Age-related increase in senescent cells was reported in the skin tissue of monkeys and humans [15, 213]. The first senolytics identified were dasatinib and quercetin; their combination treatment reduced senescent cell burden in chronologically aged, radiation-exposed, and progeroid Ercc1^−/Δ^ mice (a mouse model of a human progeroid syndrome) [214]. These results demonstrated the potential of selectively targeting senescent cells and the efficacy of senolytics for alleviating symptoms of age-related diseases and extending health span. Since then, several senolytics, such as navitoclax, 17-DMAG, and a peptide that targets the Bcl-2- and p53-related senescent cell anti-apoptotic pathways (SCAPs), have been shown to be effective in reducing senescent cells in mice as evidenced by decreasing senescent cell indicators (see reviews by Sun et al. [215] and Knoppert et al. [216] for a complete list). Following the discovery of dasatinib and quercetin, the next senolytic compound to emerge was navitoclax (ABT-263), a small molecule belonging to Bcl-2 family protein inhibitor. ABT-263 selectively binds to Bcl-2, Bcl-XL, and Bcl-w and prevents their binding to the apoptotic effectors Bax and Bak proteins [217–219]. In mouse models of atherosclerosis and neurodegeneration, ABT-263 eliminated dysfunctional senescent cells from atherosclerotic plaques and brain tissue, respectively, substantially inhibiting the progression of key disease phenotypes [220, 221]. After the discovery as a senolytic agent, ABT-263 has been extensively used to study the mechanisms of aging in several animal model systems. For example, in a model of pulmonary fibrosis, treatment of the irradiated mice with ABT-263 after persistent disease had developed reduced senescent cells and reversed the disease [222]. Fisetin [223] is another flavonoid with multipotent properties identified as a novel senolytic molecule. Acute or intermittent treatment of progeroid and old mice with fisetin reduced the biomarkers of senescence in multiple tissues, restored tissue homeostasis, reduced age-related pathology, and extended median and maximum lifespan [224]. A new class of drug candidates with senolytic properties which are inhibitors of heat-shock protein HSP90 has been discovered [225]. Multiple treatment with the HSP90 inhibitor 17-DMAG significantly delayed onset of different age-related symptoms in progeroid mice, leading to an overall health span improvement [225]. In a p16-3MR transgenic mouse model, selective depletion of senescent cells by the small-molecule chemical UBX0101 reduced the development of posttraumatic osteoarthritis by creating a proregenerative microenvironment [226]. Piperlongumine (PL) is a biologically active alkaloid and a recently identified senolytic agent which can selectively kill senescent cells by targeting oxidation resistance 1 (OXR1) to mediate PL senolytic activity [205]. The multicenter Intervention Testing Program by the National Institute for Aging has identified five drugs that increase lifespan in genetically heterogenous mice, including rapamycin, acarbose, nordihydroguaiaretic acid, 17-*α*-oestradiol, and aspirin [227]. Recent mechanistic studies show that stimulated by cytosolic DNA, active cyclic GMP-AMP synthase-stimulator of interferon gene (cGAS-STING) pathway triggers inflammation and plays a role in the development of senescence [228]. Based on these findings, it was hypothesized that blocking STING pathway could be a potential therapeutic strategy to prevent senescence-associated human diseases [229].

All the preclinical animal studies in different disease models with an array of senolytic agents provide confidence in bringing senolytic agents into clinical trials. In an open-label phase 1 pilot study, the first clinical trial of senolytics, dasatinib (D)+quercetin (Q) improved physical function in patients with idiopathic pulmonary fibrosis (IPF), a fatal senescence-associated disease and diabetic kidney disease [230, 231]. D+Q decreases p16INKA, p21, and SASP factors, including IL-1*α*, IL-6, MMP-9, and MMP-12 [230]. The senolytic drug UBX0101 was developed to treat osteoarthritis of the knee (ClinicalTrials.gov Identifier: NCT03513016). Another drug, UBX1967, a Bcl-2 family inhibitor specifically tailored for age-related diseases of the eye, including neovascular age-related macular degeneration, proliferative diabetic retinopathy, and diabetic macular edema, is also progressing to human testing [232].

However, though the initial studies regarding senolytics show positive signs, still there are concerns regarding their effectivity as therapies. For instance, it has been reported that senescent cells reappeared after cessation of senolytic treatment in a model of osteoarthritis [226]. Although a brief disruption of prosurvival pathways is sufficient to kill senescent cells, they are very effective only when administered intermittently [214]. Further, the senescence program considerably differ among cells/tissues, increasing the possibility of finding cell-/tissue-specific senolytics. With all these, targeting senescence using senolytics or by other strategies could not only ease specific diseases but also considerably improve the general health span of aged individuals.

## 16. Future Directions

The body of work cited in this review implicates the role of senescent cells in tissues from aged samples as one of the contributing causes of many age-related diseases such as lung disease, cardiovascular diseases, and arthritis, but information on the role of senescent cells in ocular diseases is sparse. There is an ongoing debate whether or not senescent cells found in aged animal models contribute in a significant way to diseases. At the same time, small-molecule senolytic drugs are being developed for treatment of age-related diseases and some drugs are under testing or in clinical trials. The following issues/questions need to be considered before senolytic drug treatment becomes a valuable tool in disease prevention.

- Senescent cells are heterogeneous, featured by different characteristics and could follow different pathways to avoid apoptosis [233]. Further, the senescent cell phenotype is dynamic and can change at the time of senescence or various points after senescence occurs. Therefore, there is no single senolytic/SASP inhibitor that could target all the senescent cells. Characterizing those heterogeneous cells using cell-specific markers using multiple approaches, such as experimental and bioinformatics, could highly advance our understanding of senescence and provide new strategies for designing therapy
- Senescent cells undergo extensive genome remodeling and changes in gene expression, which are poorly understood. Therefore, understanding the mechanisms responsible for these differences in gene expression and their role to senescence acquisition and SASPs would greatly benefit designing therapies. It is also worthwhile to explore the role of key epigenetic regulators that lead to the SASP and target them directly to prevent senescence
- Since senescent cells have many subpopulations in the same tissue, a single drug is unlikely to induce apoptosis to all of them. Therefore, in addition to senolytic drugs, SASP inhibitors, another group of molecules that show promise as treating age-related diseases, are also currently being tested. However, the components of the SASP vary based on the cell type from which senescent cells developed [234]. SASP components also vary in the presence of many drugs, and the regulation is segmental: not all SASP components are regulated, and hence SASPs could be modifiable [234]. Thus, it is important to learn how SASPs change at different stages of senescence, and how the SASP factors differ from tissue damage signals. Use of senolytic cocktail or combination therapy would be a better approach in fighting senescence-associated diseases in this regard
- It is now widely accepted from cellular studies that mitochondrial dysfunction plays a major role in senescence. Whether stress-induced or age-related mitochondrial dysfunction cause senescence *in vivo* remains to be studied in detail. Given the complexity of mitochondria and their involvement in multiple processes and functions, it is highly likely. Therefore, it is of major importance to further investigate the molecular processes behind the role of mitochondria in aging, and their potential to serve as targets for therapeutic interventions
- Multiple animal models are required to test potential senolytic drugs/senolytic cocktails and to understand the off-target effects of senolytic agents [56, 95, 102]. While genetically modified mice such as p16 transgenic mice [56] or pharmacological manipulations are available for most diseases, more work on additional models is required. In other words, it is desirable that senescence-associated disease animal models are developed in which senescent cells can be tracked *in tissues of interest*, and from which senescent cells can be cleared using the potential drugs
- Finally, research on understanding the mechanism of senescence from ocular cells and tissues is just beginning. IND-enabling studies of senolytic drugs for diseases such as AMD have recently been initiated [232]. The success of such investigations, although promising, will depend on a greater understanding of the mechanism of disease progression in the eye to develop optimal therapies that target the primary defect in the retina.

## Figures and Tables

**Table 1:** *In vitro* senescence models.

Senescence model	Abbreviation	Method of induction	References
Replicative senescence (RS)	RS	Short telomeres, linked to excess rounds of cell division	[2, 37]
Stress-induced premature senescence (SIPS)	SIPS	H_2_O_2_, t-BH, cytokines, oxidizing agents, hyperoxia, copper, UV irradiation	[37–39, 64, 65]
DNA damage response- (DDR-) induced senescence	DDR	Bleomycin or doxorubicin, gamma irradiator	[37]
Oncogene-induced senescence (OIS)	OIS	Activation and/or overexpression of oncogenes	[69, 76]
Mitochondrial dysfunction-associated senescence (MiDAS)	MiDAS	Inhibiting mitochondrial SIRT3	[74]
Epigenetically induced senescence	EIS	5-Aza-2′-deoxycytidine, sodium butyrate, trichostatin A, curcumin, C646, BRD4770	[27, 77–79]

**Table 2:** Ocular diseases associated with cellular senescence.

Diseases	Pathology	Therapeutic strategy	References
Age-related macular degeneration (AMD)	RPE senescence is associated with the pathology	N/A	[38, 86, 100]
Wet AMD	Macrophage senescence impairs cholesterol efflux and promotes neovascular AMD	N/A	[101]
Glaucoma	Senescent cells in the outflow pathway; retinal ganglion cell senescence	N/A	[90, 95, 102]
Experimental ocular hypertension	Senescence of retinal ganglion cells		[102]
Cataracts	Senescence in lens epithelial cells	NA	[103, 104]
Retinal microaneurysm	Neurons and blood vessels undergo cellular senescence in the retina	N/A	[42]
Fuchs endothelial dystrophy (FED)	Corneal endothelial cells (HCEC) senescence	Kojic acid	[105, 106]
Birdshot Uveitis	Shortening of telomere length in peripheral leukocytes.	N/A	[107]
Diabetic retinopathy	Endothelial cell senescence	N/A	[108–110]
Hyperglycemia-induced retinal microangiopathy	Senescence of the retinal microvasculature, RPE, and ganglion cell layer (GCL) type 1 diabetic rat model	N/A	[40]

**Table 3:** Mitochondrial metabolic changes in senescence.

Cell type	Source of senescence	Mitochondrial mass	Oxidative phosphorylation	Glycolysis	Mitochondrial ATP production	References
Human MRC5 fibroblasts	RS	Increased	Increased	Increased	Decreased	[172]
Human foreskin fibroblasts	RS	Increased	Increased	Increased	Increased	[169]
Human dermal fibroblasts.	RS	No change	No change	Increased	No change	[50]
Human dermal fibroblasts.	Doxorubicin	Increased	Increased	No change	Increased	[50]
Human foreskin fibroblasts	Doxorubicin	Increased	Increased	Increased	Increased	[169]
Human diploid IMR90 fibroblasts	Oncogenic *Ras*-induced senescence (OIS)	Increased	Decreased	-	Decreased	[56
Human Dermal Fibroblasts	Oncogene-induced senescence (OIS)	NA	Increased	-	-	[167]
Human fetal lung fibroblasts	Oncogene-induced senescence (OIS)	-	Increased	-	-	[166]
Human fibroblasts	Radiation induced	-	No change	Increased	-	[173]
Mouse melanoma B16-F1 cell line	Temozolomide (genotoxic agent)	Increased	Increased	Decreased	increased	[174]
HEI-OC1 auditory cells	SIPS (H_2_O_2_)		Decreased	-	No change	[175]
Human lung fibroblasts	SIPS (H_2_O_2_)	-	Increased	-	Increased	[176]
Human lung and cardiac fibroblast cells	SIPS (nucleoside analogs)	Increased	Increased	-	Increased	[177]
Human disc cells	SIPS	Increased	Increased	-	Increased	[168]
Human fibroblasts	*γ* radiation	-	No change	Increased	-	[173]

RS: replicative senescence; OIS: oncogene-induced senescence; SIPS: stress-induced premature senescence.
